# Quantitative Protein Profiling of *Chlamydia trachomatis* Growth Forms Reveals Defense Strategies Against Tryptophan Starvation*[Fn FN2]

**DOI:** 10.1074/mcp.M116.061986

**Published:** 2016-10-26

**Authors:** Ole Østergaard, Frank Follmann, Anja W. Olsen, Niels H. Heegaard, Peter Andersen, Ida Rosenkrands

**Affiliations:** From the ‡Department of Autoimmunology and Biomarkers, Statens Serum Institut, DK 2300 Copenhagen, Denmark;; §Department of Infectious Disease Immunology, Statens Serum Institut, DK 2300 Copenhagen, Denmark

## Abstract

*Chlamydia trachomatis* is one of the most common sexually transmitted bacterial pathogens in humans. The infection is often asymptomatic and can lead to chronic manifestations. The infectious elementary body and the replicating reticulate body are the two growth forms in the normal developmental cycle. Under the influence of interferon-γ, the normal cycle is disrupted because of tryptophan degradation, leading to a third persistent form, the aberrant reticulate body.

For the genital strain *C. trachomatis* D/UW-3/CX we established a quantitative, label-free proteomic approach, and identified in total 655 out of 903 (73%) predicted proteins, allowing the first quantitative comparison of all three growth forms. Inclusion membrane proteins and proteins involved in translation were more abundant in the reticulate body (RB)^1^ and aberrant reticulate body (ARB) forms, whereas proteins of the type III Secretion System and the cell envelope were more abundant in the elementary body (EB) form, reflecting the need for these proteins to establish infection and for host interactions.

In the interferon-γ induced ARB proteome, the tryptophan synthase subunits were identified as biomarkers with a strong increase from less than 0.05% to 9% of the total protein content, reflecting an inherent defense strategy for the pathogen to escape interferon-γ mediated immune pressure. Furthermore, the total tryptophan content in the ARB form was 1.9-fold lower compared with the EB form, and we demonstrate that modulation of the protein repertoire toward lower abundance of proteins with high tryptophan content, is a mechanism which contributes to establish and maintain chlamydial persistence. Thus, quantitative proteomics provides insights in the Chlamydia defense mechanisms to escape interferon-γ mediated immune pressure.

Chlamydia trachomatis is the causative agent of trachoma (blinding disease, ocular serovars A-C), sexually transmitted disease (genital serovars D-K) or lymphogranuloma venereum (strains L1-L3). There are about 100 million new cases of sexually transmitted *C. trachomatis* each year which can lead to pelvic inflammatory disease and infertility (1). *C. trachomatis* may be treated by single-dose antibiotics, but in many cases the infection is asymptomatic and remains undiscovered. Chronic manifestations of the infection (2) and Chlamydia-induced arthritis (3) have been linked to persistent forms of *C. trachomatis*, but it is still not understood to what extent the persistence phenotype plays a role in the Chlamydia induced pathology.

*C. trachomatis* is an obligate intracellular pathogen with a small genome encoding 895 ORFs and most strains in addition contain an extrachromosomal plasmid with eight ORFs (4). *C. trachomatis* has adapted to intracellular parasitism, *i.e.* enzymes and pathways are missing in the small genome (4), and the pathogen is instead dependent on the host providing metabolic intermediates including amino acids. *C. trachomatis* has a unique developmental cycle that starts when an infectious elementary body (EB) enters an epithelial cell via endocytosis. Inside the inclusion vacuole, the EB rapidly differentiates into an RB that is noninfectious but metabolically active and can undergo replication. The progeny RBs differentiate back into EBs, which eventually exit the infected cell for spreading to new host cells and initiate another cycle (supplemental Fig. S1).

Chlamydia infection leads to a cellular immune response and secretion of interferon-γ (IFN-γ) in the human host. In the epithelial cells, secreted IFN-γ leads to induction of indoleamine 2,3-dioxygenase (IDO), which degrades the essential amino acid tryptophan (Trp), and this causes Trp deprivation for the Trp auxotroph *C. trachomatis*. The normal developmental cycle will then be disrupted and lead to development of a persistent form, the nonreplicating aberrant reticulate bodies (ARB), which are viable, but noncultivable (supplemental Fig. S1). Using tryptophan synthase, genital strains of *C. trachomatis* may synthesize Trp from indole provided by the microflora in the female genital tract (5). If Trp is added, the ARB form can reactivate to enter the normal growth cycle (6). Transcriptome analysis of *C. trachomatis* in an *in vitro* model of IFN-γ-mediated persistence demonstrated up-regulation of the tryptophan synthase genes, *trpA* and *trpB*, arrest of RB to EB transition and blocking of cell division, and suggested IFN-γ-induced persistence as a defense strategy to avoid the host immune response (7). Interestingly, a recent study observed the ARB phenotype in a human endocervix sample and it was further characterized by lower indole levels and inclusion forming units, but normal genome copy numbers, a high IFN-γ response, and a high *euo/omcB* mRNA ratio (8).

Other *in vitro* conditions which induce persistence have been described, as reviewed by Wyrick and Hogan *et al.* (2, 9). These inducers include antibiotics, iron deprivation, nutrient (amino acid) starvation, co-infection with herpes simplex or monocyte infections.

Proteome analysis of the *C. trachomatis* growth forms is challenging because of a mixed-proteome situation where host cell proteins copurify with the bacterium. In early studies, purified Chlamydia bacteria were analyzed by two-dimensional gel electrophoresis and protein spots were identified by MALDI-TOF after ^35^S-labeling of bacterial proteins (10, 11) or by different sequential LC-MS/MS techniques (12). Saka *et al.* applied LC/LC-MS/MS for analysis of the *C. trachomatis* L2 proteome, which also allowed a label-free quantitation of the 485 identified proteins (13). The study demonstrated that varying amounts of human proteins derived from the host cells (HeLa cells) copurify with the *C. trachomatis* bacteria and highlighted the need for adjustments when quantitative comparisons between the RB form and the EB form were undertaken. Recently, Skipp *et al.* characterized the EB and RB proteomes of L2 as a model strain for *C. trachomatis* using 2D-RPLC-MS. The study identified 562 proteins of which 556 have orthologs in the D/UW-3/CX proteome (14).

The previous LC-MS/MS studies have focused on the *C. trachomatis* L2 strain, which is superior as a model strain in the laboratory, but the strain is the cause of the invasive form of disease, not the urogenital form. Here, we investigated the *C. trachomatis* D/UW-3/CX strain as a model strain for urogenital Chlamydia infections. Furthermore, for the first time, we quantitatively analyzed the proteomes and the Trp content of the persistent IFN-γ induced ARB forms as well as the RB and EB forms and thus cover all known growth forms.

## EXPERIMENTAL PROCEDURES

### 

#### 

##### Chlamydia Cultures

HeLa 229 cells were grown in RPMI medium supplemented with 5% fetal calf serum at 37 °C and 5% CO_2_. Cells were seeded in six-well plates at 5 × 10^5^ cells per well and the monolayers were infected by centrifugation after 24 h with *C. trachomatis* serovar D/UW-3/CX at a multiplicity of infection of 4 (15). Infected cells were incubated in the absence or presence of IFN-γ (2 ng/ml equaling 40 IU/ml). Triplicate samples of the RB, EB, and IFN-γ induced ARB forms (each originating from 12 plates) were harvested after 20, 40, and 40 h post infection, respectively, by scraping infected HeLa cells from the six-well plates. After sterile filtration, the Trp content in the spent culture medium was determined by the Bridge-It® l-Trp assay according to the manufacturer's instructions (Mediomics, LLC, St. Louis, MO).

##### Antibodies

Antibodies against MOMP, OmcB, TrpA, TrpB, RpoB (amino acids 2–384), Euo (amino acids 2–183), IncG (amino acids 5–33 and 89–167), PmpG (amino acids 28–387), and PmpH (amino acids 25–387) were raised by immunizing rabbits with recombinant His-tagged proteins adjuvanted with Montanide ISA 720 (Seppic, Puteaux Cedex, France). Anti-human IDO was obtained from Genecust (Dudelange, Luxembourg) after conjugation of the peptide LEAKGTGGTDLMNFLKTVRSTTEKSLLKEG to Keyhole limpet hemocyanin and immunization of rabbits. MOMP, OmcB, TrpA, TrpB, and IDO antisera were purified by Protein G column chromatography and labeled with either Alexa Flour 488 or 568 according to the manufacturer's instructions (Life Technologies, Carlsbad, CA).

##### Microscopy

LAB-TEK® chamber slides (4 wells) were seeded with HeLa cells (1.5 × 10^5^ cells per well) in RPMI medium supplemented with 5% fetal calf serum and infected after 24 h with *C. trachomatis* serovar D/UW-3/CX, either in the presence or absence of IFN-γ (2 ng/ml). After 20 or 40 h of infection, the medium was removed and the cells were fixed with 99% ethanol for 10 min and washed with PBS. Blocking was performed with 1% BSA in PBS, followed by incubation with Alexa Flour 488 or 568 labeled antibodies diluted in 0.1% BSA in PBS. Slides were finally mounted with mounting solution containing DAPI (Fluorogel with DAPI, Electron Microscopy Services, Hatfield, PA). The infected cells were visualized by a Confocal Microscope (Zeiss LM710, Jena, Germany).

##### Preparation of Protein Extracts and Sample Preparation

Purification of RB, EB, and ARB forms was performed by homogenizing the harvested HeLa extracts in K-36 buffer (0.05 m potassium phosphate, 0.1 m potassium chloride, 0.15 m sodium chloride, pH 7.0) with a Wheaton Dounce Homogenizer as described (16). Briefly, host material was removed by centrifugation at 500 × *g* (15 min) and bacteria were collected at 35,000 × *g* (30 min). RB, EB, and ARB forms were further purified on Renografin solutions and ultracentrifugation (40,000 × *g*, 30 min). The pellets were resuspended in SPG buffer (0.25 m sucrose, 10 mm sodium phosphate, 5 mm glutamic acid, pH 7.4), and centrifuged (35,000 × *g*, 30 min). Pellets were resuspended in 1 ml 2% SDS, 50 mm ammonium bicarbonate, 5 mm DTT (pH 7.8), and sonicated 4 × 30 s on ice. The lysates were centrifuged (16,000 × *g*, 20 min), and the supernatants were collected for the proteomic analyses. The total protein content in the samples was determined by the 2D-Quant method (GE Healthcare, Uppsala, Sweden).

The chlamydial lysates were analyzed by SDS-PAGE followed by LC-MS/MS (17). Briefly, 50 μg of total protein was prepared from each sample by 2D Clean-up kit (GE Healthcare). The proteins were separated by SDS-PAGE using precast 4–12% gradient gels (NuPAGE MES system, Invitrogen, Carlsbad, CA), and the gels were stained with Novexin Instant Blue (Expedeon Protein Solutions Ltd, Cambridge, United Kingdom). For each Chlamydia growth form three SDS-PAGE lanes representing three biological replicates of each sample type were cut into 10 bands which were subjected to in-gel trypsin digestion (18). The peptides formed by the digestion were dried on a vacuum concentrator, reconstituted in 5% formic acid, and desalted on pre-equilibrated homemade StageTips (19).

##### LC-MS/MS Analysis

Peptides were loaded onto an Acclaim PepMap C18 precolumn (300 μm inner diameter, 5 mm long, 5 μm particle size, Dionex, Hvidovre, Denmark), and separated using an Acclaim PepMap100 C18 analytical column (75 μm inner diameter, 150 mm long, 3 μm particle size, Dionex) by a 90-min gradient controlled by a Dionex Ultimate 3000 nano-LC system connected to an LTQ Orbitrap XL mass spectrometer (Thermo Fisher Scientific, Waltham, MA) equipped with a nano-electrospray source (Proxeon, Odense, Denmark). The flow rate was 200 nL/minute; the mobile phases consisted of solvent A (2% (v/v) acetonitrile, 0.1% (v/v) formic acid) and solvent B (95% (v/v) acetonitrile, 0.1% (v/v) formic acid). The gradient went from 0% to 45% solvent B in 65 min, followed by 25 min with 100% solvent B; then data acquisition was stopped and the column was re-equilibrated with solvent A.

MS data were acquired recording full scan spectra (250–1800 mass/charge (*m*/*z*)) in the Orbitrap with 60,000 resolution at 400 *m*/*z*. MS/MS data were recorded in parallel in a data-dependent mode, fragmenting the five most abundant ions (charge state +2 or higher) by collision-induced dissociation in the LTQ ion trap at 35% collision energy. MS/MS spectra were recorded using dynamic exclusion (30 s) to minimize repeated fragmentation of the same peptides.

Recorded raw files were analyzed using MaxQuant version 1.2.2.5 (20) for peptide quantitation by MS1-intensity and for protein identification using the built-in Andromeda search engine (21). In addition, intensity-based absolute quantification (iBAQ) values were calculated (22) for ranking of the absolute abundance of different proteins within a single sample. The MaxQuant analysis was performed with the following settings: Enzyme: Trypsin, and maximum 1 missed cleavage. Precursor mass tolerance was 6 ppm, and fragment mass tolerance 0.5 Da. Variable modifications: Oxidation (M), and acetyl (protein *N*-terminal). Fixed modifications: Carbamidomethyl (C). Multiplicity: 1 (no isotope labeling). Peptide FDR 1%, protein FDR 1%, minimum peptides: 1. Match between runs 1 min. Keep low-scoring version of identified peptides: On. All other settings were left at the default settings.

Proteins were identified by peptide-spectral matching searching against the following sequence files in FASTA format: *Chlamydia trachomatis* genome (NC_000117_D/UW-3/CX) including 895 sequences in amino acid format—downloaded from NCBI genomes 2015–03-20, *Chlamydia trachomatis* D-LC plasmid ORFs (8 entries) downloaded 2014–03-04, *Homo sapiens* UniProt reference proteome downloaded from UniProt 2014–01-07 with 69025 entries, and a FASTA-file accompanying MaxQuant containing common contaminants. Moreover, MaxQuant was set to automatically generate a decoy FASTA-file with the reversed sequences of the above files to estimate false positive peptide spectral matches using a target-decoy strategy (23). Protein tables were filtered to eliminate the identifications from the reverse database and common contaminants.

The mass spectrometry proteomics data (raw data and search results files generated by MaxQuant) have been deposited to the ProteomeXchange Consortium (http://proteomecentral.proteomexchange.org) with the data set identifier PXD003883.

All protein IDs reported and discussed in the presented work are based on two unique peptides; for reference, protein identifications based on only 1 peptide (31 proteins) are included in the deposited raw data and in the supplemental Data S1 file.

##### Data Analysis

Protein MS1 intensities were used to compare protein abundances for individual proteins across samples whereas iBAQ values were applied for ranking abundances of all identified proteins within individual samples. MS1 intensities and iBAQ values for a given sample were normalized by the ratio of the average total intensity of all samples and the total intensity of the individual sample.

Venn diagrams were constructed using Venny 2.0 (http://bioinfogp.cnb.csic.es/tools/venny/index.html) to illustrate how the identified proteins were distributed among the analyzed fractions.

##### Functional Enrichment Analysis

The identified proteins were annotated in functional categories based on the information available at http://stdgen.northwestern.edu, and supplemented according to results from the literature (24–28), KEGG (http://www.genome.jp/kegg/), Uniprot (http://www.uniprot.org/) and NCBI http://www.ncbi.nlm.nih.gov/protein/). Unknown, uncategorized, hypothetical, and “other category” proteins were grouped together as uncategorized proteins. The minimal size of the categories evaluated was set to 5 proteins.

##### Immunoblot Analysis

Mini-PROTEAN® TGX™ 4–20% precast gels (BIO-RAD, Hercules, CA) were used, and after electrophoresis protein extracts were transferred to nitrocellulose. The membranes were blocked with 5% skimmed milk powder (SMP) in PBS-T (PBS, 0.1% Tween 20, pH 7.4), followed by incubation with rabbit sera raised against: MOMP, OmcB, TrpA, TrpB, RpoB, Euo, IncG, PmpG, and PmpH. The primary antibodies were diluted 500-fold in 1% SMP in PBS-T and the horseradish peroxidase conjugated goat-anti-rabbit secondary antibodies (Thermo Fisher Scientific) were diluted 1000-fold in 1% SMP in PBS-T. Chemiluminescence detection was performed with ECL Prime reagent (GE Healthcare) on a G:BOX Chemi XX6 instrument.

##### Trp Content Calculations

The Trp content of a given protein was calculated based on the number of Trp residues *versus* the total number of amino acids in the amino acid sequence of the protein as downloaded from http://www.uniprot.org/. The molar equivalents of Trp for a given protein was for each sample calculated as the number of Trp residues for the protein multiplied with the normalized protein iBAQ value of the sample.

##### Experimental Design and Statistical Rationale

The total numbers of biological samples analyzed by LC-MS/MS analysis were three of all sample types RB, EB, and IFN-γ induced ARB to allow a quantitative comparison. The samples were further analyzed in technical duplicates.

Quantitative comparisons were performed for identified proteins which were present in at least two out of three replicates from either sample type (RB, EB, or ARB). Relative protein abundance differences were analyzed individually for each protein by one-way analysis of variance (ANOVA) with Tukey post-hoc test by the XLSTAT program (a statistical analysis add-in to Microsoft Excel) after log transformation of the normalized MS1 intensity values. To account for zero intensity values one-tenth the minimum nonzero value was imputed for that specific protein. Significantly changed proteins were identified using a fold change of >2 or <0.5 and *p* value of <0.05 after Benjamini-Hochberg correction for multiple testing. Fold change was calculated as RB/EB, ARB/EB, and RB/ARB ratios of mean normalized MS1 intensity values using data imputation as described above for zero values.

In the functional enrichment analysis, overrepresented functional categories for differentially abundant proteins were determined by the Fisher's exact test using GraphPad Prism software version 6. A *p* value of less than 0.05 was considered significant.

Comparison of the Trp content in proteins with changed abundance was performed by the Kruskal-Wallis test with Dunn's post test using GraphPad Prism. A *p* value of less than 0.05 was considered significant.

## RESULTS AND DISCUSSION

### 

#### 

##### The In Vitro Model of Chlamydial Infection

**Fig. 1.**
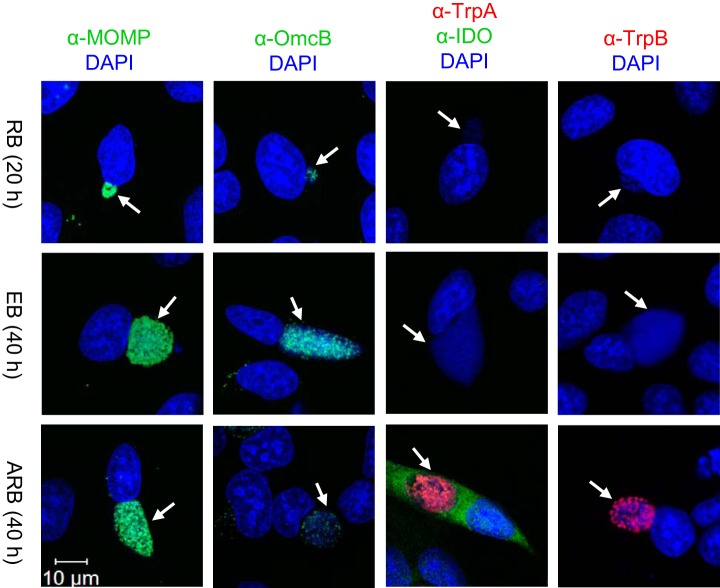
**Confocal microscopy images of HeLa cells infected with *C. trachomatis* serovar D/UW-3/CX cultured in supplemented RPMI medium in the absence (RB, EB) or presence of IFN-γ (ARB).** Either 20 or 40 h post infection cells were fixed and stained with antibodies for MOMP (*green*), OmcB (*green*), IDO (*green*), TrpA (*red*) or TrpB (*red*), as well as DAPI (*blue*) to detect host and bacterial DNA. *White arrows* indicate the position of inclusions and the *white scale bar* indicates 10 μm.

We established the conditions for studying *C. trachomatis* in the RB, EB, and IFN-γ induced ARB growth forms by infecting HeLa 229 cells with the reference strain D/UW-3/CX (originally isolated from the cervix). Time points for studying RB, 20 h, and EB, 40 h, were selected to obtain the highest yield and purity of the relevant growth form (29). ARBs were induced by adding IFN-γ to the HeLa cells at the time of infection with *C. trachomatis*. The *in vitro* model was characterized by confocal microscopy of infected HeLa cells harboring the RB, EB, and ARB forms (Fig. 1). The major outer membrane protein (MOMP, encoded by the *ompA* gene) was detected in all growth forms, whereas the cysteine-rich, outer membrane complex protein B (OmcB) was predominant in the EB form. IDO is a host marker for IFN-γ-mediated persistence, and *C. trachomatis* TrpA and TrpB, the tryptophan synthase A and B components, are bacterial proteins induced upon IFN-γ-treatment (30). IDO staining of *C. trachomatis* infected HeLa cells was only detected in the presence of IFN-γ, and TrpA and TrpB were exclusively detected in the ARBs, highlighting the potential of these proteins as biomarkers for IFN-γ-induced persistence. The Trp levels determined in the spent medium from the cultures confirmed the decrease of Trp in the ARB cultures and furthermore showed that the transition from RB to EB form required a substantial amount of Trp from the culture medium (supplemental Fig. S2).

##### The Chlamydia trachomatis Proteomes

For proteomic analysis, bacterial pellets were isolated from infected HeLa cells. Initial LC-MS/MS analysis of the tryptic peptide mixtures showed a higher proportion of chlamydial proteins relative to host protein in the EB samples in agreement with previous results (13). As a “mixed proteome,” it is not possible in the HeLa-*C. trachomatis* system to isolate the bacteria without varying amounts of host cell proteins.

**Fig. 2.**
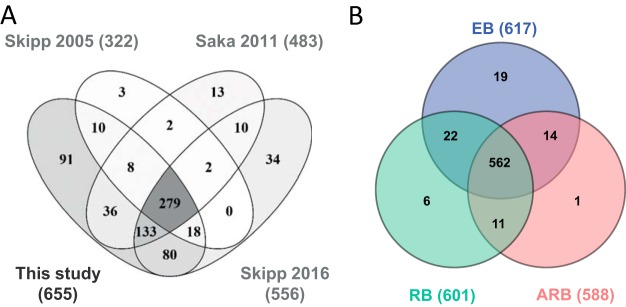
**Identification of the *C. trachomatis* proteome.** *A*, Venn diagram of the number of proteins from the D/UW-3X/CX strain identified in this study by a minimum of two peptides compared with previous studies on the L2 strain (12–14). *B*, Venn diagram showing the overlap of the proteins identified and quantified in the RB, EB, and ARB forms, in total 635 proteins. Proteins were selected if they were identified in at least two of the triplicate samples from either growth form.

To perform an unbiased quantitative comparison of the chlamydia protein levels across growth forms, the EB samples were spiked with HeLa protein lysate to obtain the same level of host proteins and thereby allow reliable comparisons of the abundancies of *C. trachomatis* proteins in all samples. Based on at least two unique peptides, we identified 3424 proteins HeLa proteins (*H. sapiens*) after SDS-PAGE separation, tryptic digestion, and LC-MS/MS analysis. Likewise, we identified 655 *C. trachomatis* proteins corresponding to 73% of the proteins predicted from the chlamydia genome sequencing project (4) in the samples of RB, ARB, EB before spiking (in short EB_pre_) and EB spiked with HeLa (EB) (supplemental Data S1 and supplemental Table S1). Only 42% of the predicted Chlamydia proteins with a mass below 10 kDa were identified, whereas at least 65% of the genome predicted proteins were identified from the mass regions above 10 kDa (supplemental Fig. S3*A*), which may reflect the general challenge in the identification of low mass proteins (31). More than 99% of the identified proteins had calculated pI values in the range 4–12, and we did not observe underrepresentation of acidic or basic proteins (supplemental Fig. S3*B*) as has been reported for studies where 2D PAGE is used as separation step (32). The initial SDS-PAGE separation step allowed a higher amount of starting material and less complex peptide mixtures to be analyzed in each run by the LC-MS/MS system, and led to identification of 91 novel *C. trachomatis* proteins in this study compared with previous proteome studies (12–14) (Fig. 2*A*).

**Fig. 3.**
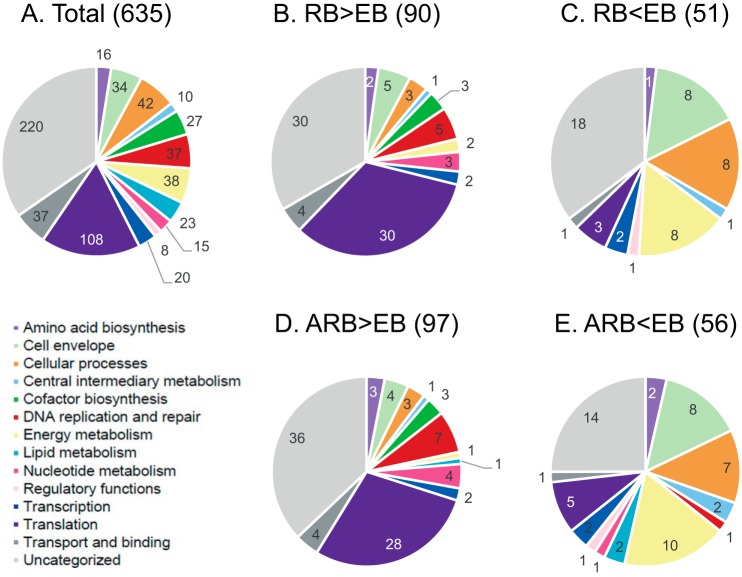
**Pie charts representing functional categorization of identified *C. trachomatis* proteins.** *A*, Total list of proteins quantified by LC-MS/MS for comparison of RB, EB and ARB forms. *B*, Proteins in the RB form with increased abundance and (*C*) decreased abundance compared with the EB form. *D*, Proteins in the ARB form with increased abundance and (*E*) decreased abundance compared with the EB form. The number of proteins belonging to each category is shown.

For further comparison of the RB, EB, and the IFN-γ induced ARB samples, we selected the 635 proteins which were identified in at least two out of three replicates from either sample type (RB, EB, or ARB; supplemental Table S2). The overlap in protein identifications from the three growth forms is shown in Fig. 2*B*. Notably, most proteins (562 of 635, 89%) were shared between all three forms. This is in agreement with the assumption that virtually all genes of the small genome of *C. trachomatis* are expressed during the developmental cycle. An overview of all 635 proteins according to functional category is presented in Fig. 3*A*, primarily based on the functional classes annotated by the Los Alamos National Laboratory Bioscience Division (http://stdgen.northwestern.edu/); one third of the proteins do not have an assigned function. In agreement with the expected expression of nearly all predicted genes, the functional distribution of the identified proteins appeared quite similar for the RB, EB, and ARB forms (supplemental Fig. S4).

##### Quantitative C. trachomatis Protein Profiling

A quantitative analysis was then undertaken using normalized iBAQ values to estimate the individual protein abundances within each growth form. The abundances were calculated as the sum of iBAQ values from all gel slices, but in almost all cases peptide intensities peaked in one gel slice (data not shown). For the majority of the identified proteins this quantification approach was expected to be adequate, but it should be emphasized that if a protein was represented in multiple protein species, they may not be distinguished by the strategy applied here.

**Fig. 4.**
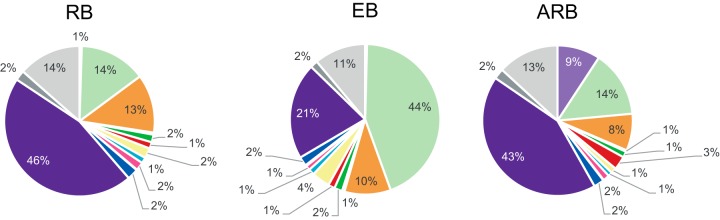
**Quantitative distribution of functional groups in the RB, EB and ARB forms based on intensity-based absolute quantification (iBAQ) values.** For each growth form, the mean total molar equivalents of each category was calculated based on the three biological replicates. The colors used for functional categories are the same as in Fig. 3.

The ten most abundant proteins in RB, EB, and ARB are shown in Table I. MOMP (Ct681) was the most abundant protein in all growth forms and represented 39.1% of the quantitated proteins from the EB form *versus* about 13% in the RB and ARB forms. Other common abundant proteins were the heat shock proteins GroEL and GroES (Ct110 and Ct111), and the elongation factor Tuf. Proteins found abundant only in the EBs were OmcB, the histone-like protein HctA and the dihydroneopterin aldolase FolB. MOMP, and OmcB are highly disulfide cross-linked in EBs and play a role in the structural stability of the bacteria under osmotic stress (33). Bacterial chromatin condensation is a distinctive feature of the EB form and is caused by histone-like proteins such as HctA. FolB is involved in *de novo* biosynthesis of folate cofactors required for DNA and amino acid synthesis (34). The thiol specific antioxidant AhpC is involved in protection against oxidative damage, and was among the 10 most abundant proteins in both the EBs and RBs. Ribosomal proteins (RplM, Rply, RplV, RpsD, RpsM) were abundant in the RBs and ARBs in agreement with the up-regulation of genes involved in translation in these growth forms (7, 35). Finally, TrpA and TrpB, representing 9% of total ARB protein, ranked as the second and fourth most abundant proteins in this growth form, while being absent from the top 10 list of EB and RB quantified proteins where they only constituted 0.03 and 0.05% of the total protein, respectively. This reflects a strongly up-regulated expression of the corresponding genes during Trp depletion (7). In Fig. 4 the quantitative distribution of all the identified proteins is shown according to functional category. Translation was the top category in the RB and ARB samples (46 and 43%), and only in the ARBs the amino acid biosynthesis category was substantial because of the increased abundance of TrpA and TrpB. In the EBs, the cell envelope was the top category accounting for 44% of the quantified proteins.

**Table I:** The most abundant proteins identified in EB, RB, and ARB growth forms

	Ct No	Gene	iBAQ ± S.D.*^a^*	Abundance (%)*^b^*
RB	Ct681	*ompA*	(2.21 ± 0.39) × 10^8^	13.1
	Ct322	*tuf*	(9.13 ± 2.27) × 10^7^	5.4
	Ct110	*groL*	(7.59 ± 3.57) × 10^7^	4.5
	Ct603	*ahpC*	(4.91 ± 0.38) × 10^7^	2.9
	Ct111	*groS*	(3.98 ± 2.41) × 10^7^	2.3
	Ct691	*-*	(2.71 ± 0.26) × 10^7^	1.6
	Ct509	*rpsM*	(2.71 ± 0.14) × 10^7^	1.6
	Ct126	*rpsI*	(2.63 ± 0.23) × 10^7^	1.5
	Ct125	*rplM*	(2.34 ± 0.30) × 10^7^	1.4
	Ct799	*rplY*	(2.32 ± 0.60) × 10^7^	1.4
EB	Ct681	*ompA*	(6.62 ± 0.73) × 10^8^	39.1
	Ct443	*omcB*	(5.69 ± 0.88) × 10^7^	3.4
	Ct322	*tuf*	(5.34 ± 0.76) × 10^7^	3.1
	Ct110	*groL*	(3.65 ± 1.08) × 10^7^	2.1
	Ct659	*-*	(3.30 ± 1.85) × 10^7^	1.9
	Ct603	*ahpC*	(2.61 ± 0.32) × 10^7^	1.5
	Ct111	*groS*	(2.31 ± 0.42) × 10^7^	1.4
	Ct743	*hctA*	(1.75 ± 0.69) × 10^7^	1.0
	Ct043	*-*	(1.37 ± 0.39) × 10^7^	0.8
	Ct614	*folB*	(1.19 ± 0.67) × 10^7^	0.7
ARB	Ct681	*ompA*	(2.19 ± 0.73) × 10^8^	12.9
	Ct171	*trpA*	(9.80 ± 2.98) × 10^7^	5.8
	Ct322	*tuf*	(7.16 ± 2.74) × 10^7^	4.2
	Ct170	*trpB*	(5.22 ± 1.58) × 10^7^	3.1
	Ct110	*groL*	(3.79 ± 1.05) × 10^7^	2.2
	Ct799	*rplY*	(3.34 ± 0.72) × 10^7^	2.0
	Ct626	*rpsD*	(3.26 ± 1.06) × 10^7^	1.9
	Ct523	*rplV*	(3.11 ± 0.72) × 10^7^	1.8
	Ct111	*groS*	(2.44 ± 0.78) × 10^7^	1.4
	Ct691	*-*	(2.40 ± 0.06) × 10^7^	1.4

*^a^* Mean normalized iBAQ values are shown.

*^b^* The relative abundance is shown.

To compare the individual protein abundances across samples, normalized MS1 intensity values were analyzed, and the coefficient of variances (CV's) calculated for all identified proteins in each growth form (supplemental Fig. S5). One-way ANOVA analysis on the log transformed MS1 intensity values was performed, and proteins which displayed significant change (> 2-fold difference, *p* < 0.05 after Benjamini-Hochberg correction for multiple testing) were selected. We identified 90 proteins with significantly increased abundance in the RBs compared with the EBs and 51 proteins with decreased abundance (supplemental Table S2). The functional distribution of the proteins with changed abundance in the RBs is presented and compared with the profile of all 635 identified proteins (Fig. 3*A*–3*C*). Likewise, in the ARBs, 97 proteins had significantly increased abundance and 56 decreased abundance compared with the EBs (Fig. 3*A*, 3*D*–3*E*, supplemental Table S2).

##### The Proteomes of the Developmental Cycle Growth Forms

**Fig. 5.**
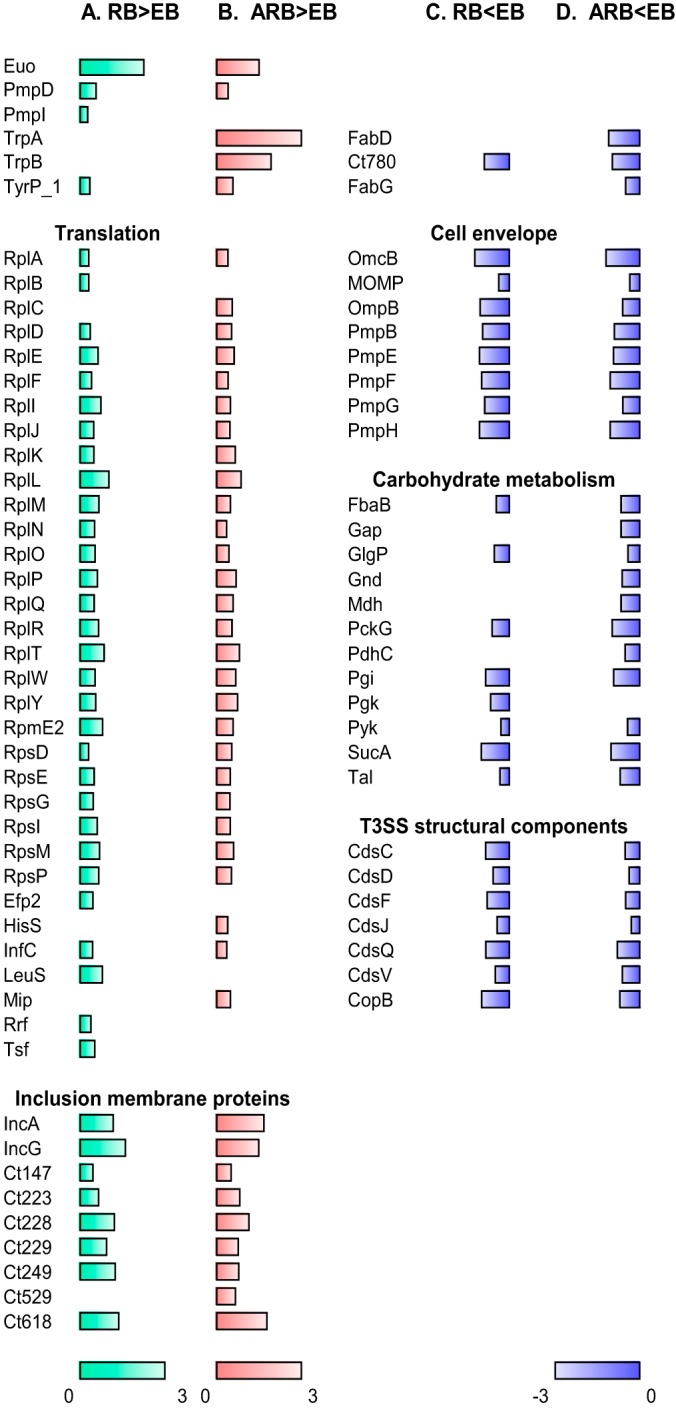
**Overview of selected proteins that display changed abundance (fold change >2, *p* < 0.05).** *A*, Proteins with increased abundance in the RB form compared with the EB form, *B*, increased abundance in the ARB form compared with the EB form, *C*, decreased abundance in RB compared with EB, *D*, and decreased abundance in ARB compared with EB. The *bars* depict the log_10_ transformed fold change values; the *green bars* represent RB/EB abundance ratio values between 0 and 3, the *red bars* ARB/EB ratio values between 0 and 3, and the *blue bars* RB/EB or ARB/EB ratio values between −3 and 0. *Bars* are only shown for proteins with changed abundance. The complete data set is listed in Supplemental Table 2.

To further compare the RB and EB proteomes we looked for overrepresentation of changed proteins in the associated functional categories. Translation was identified as an enriched functional category in RBs (Fig. 5*A*, supplemental Table S3). In addition, the group of uncategorized proteins included the inclusion membrane proteins (Inc proteins), as described (24, 25, 28). We found enrichment of this group represented by IncA, IncG, Ct147, Ct223, Ct228, Ct229, Ct249, and Ct618 previously described as encoded by “early genes” (35), and confirmed the presence of these proteins predominantly in the RB form. Furthermore, we identified three other proteins previously described to be more abundant in the RBs: Euo is a repressor which regulates the late gene expression (36) and polymorphic membrane protein D (PmpD) is a virulence factor important for the early attachment to the human cell (37). The finding that PmpD and PmpI were more abundant in RBs compared with the EBs confirms observations from the L2 strain (13).

Overrepresented protein categories with decreased abundance in the RBs represented the cell envelope subcategory membranes, lipoproteins, and porins including the most abundant outer membrane proteins MOMP and OmcB, proteins involved in carbohydrate metabolism according to KEGG, and structural components of the type III secretion system (T3SS) (Fig. 5*C*, supplemental Table S3). These findings reflect the formation of the outer membrane complex in the EB form and the ability of the EBs to synthesize ATP through carbohydrate metabolism, in contrast to the RBs, which depend on ATP from the host cell (38). Moreover, the EBs are thought to be preloaded with the structural components of the T3SS in order to translocate proteins involved in host interactions early in a new infection cycle (39). Other proteins decreased in the RBs included the Ct780 disulfide isomerase predicted to be involved in the formation of the disulfide cross-linked outer membrane complex in the EBs (35).

Our overall findings of increased T3SS capacity and carbohydrate metabolism in the EB form are in contrast to the recent study in the L2 strain (14), but support previous observations from the L2 strain (13). The reasons for the discrepancy between the two studies investigating the same invasive strain are not known.

##### The Proteome of Chlamydial Persistent Infection

The most striking characteristics of the IFN-γ induced ARB form compared with the EB form was the exceptionally strong increase in the abundance of both TrpA and TrpB (1060 and 87 average fold change, *p* < 0.0001 and *p* = 0.0026, respectively). The tryptophan synthase enables synthesis of Trp from indole, a compound that is not present in our *in vitro* system, but it may be present in the microenvironment produced by the vaginal microbial flora (5). Increase in the tryptophan transporter TyrP_1 permease was also observed, emphasizing the need for import of Trp from the host to the inclusion. Translation associated proteins were overrepresented among the proteins with increased abundance in the ARB form, as also observed for the RBs (Fig. 5*B*, supplemental Table S3). Furthermore, Inc proteins were identified as an overrepresented category of the ARB-increased proteins, which fits well with the previous gene expression study (7). Heat shock proteins have frequently been linked to immunopathologic manifestations of Chlamydia infections (40, 41). We identified 2 of the 3 hsp60 gene products, Ct110 and Ct755, but none of them showed increased abundance in the ARB form, in agreement with previous *in vitro* studies (35, 42).

Proteins decreased in ARB were within the categories cell envelope subcategory membranes, lipoproteins, and porins, carbohydrate metabolism, and structural components of the T3SS (Fig. 5*D*, supplemental Table S3). This confirms the downregulation of late genes involved in formation of the outer membrane complex as reported in the transcriptomics study of persistence (7) and decreased expression of genes related to energy metabolism, as was observed in previous persistence studies (35, 43). The predicted disulfide isomerase Ct780 and the two enzymes involved in fatty acid biosynthesis, FabD and FabG, were also less abundant in the ARB form. Down regulation of late genes, carbohydrate metabolism, and fatty acid biosynthesis pathways is compatible with a persistence phenotype restricted in nutrients and inhibited in the differentiation to the EB form.

To confirm the MS-based protein quantitation methods used in this study, the three different growth forms were also characterized by immunoblots using antibodies raised against *C. trachomatis* proteins expected to be present either in all growth forms or to display differential abundance (supplemental Fig. S6). The RNA polymerase beta subunit RpoB protein was detected in similar amounts across all samples, both in immunoblots and by normalized MS1 intensity values, in agreement with the “housekeeping” status of this protein. The IncG protein was more abundant in the RB and ARB forms, and the Euo repressor was most abundant in the RBs. MOMP was present in all growth forms, although mostly abundant in the EB form. OmcB, PmpG, and PmpH were highly enriched in the EBs as also previously observed (7, 13, 35). Finally, TrpA and TrpB were almost exclusively detectable in the ARB form. Overall, we observed a high degree of consistency between the normalized MS1 intensity and the immunoblot band intensities.

In summary, we observed that the protein profiles of RB and ARB forms appear quite similar, except for the strong increase in TrpA and TrpB observed in the ARB form. This probably reflects that chlamydial proteins are quite stable in order to maintain the viability of the persistent form as previously suggested for *C. pneumoniae* (44). The ANOVA analysis identified 18 proteins with increased abundance in the ARB form compared with the RB form and 21 proteins with decreased abundance (supplemental Table S2, supplemental Fig. S7). However, no overrepresented functional categories were observed for the ARB-increased or -decreased proteins compared with the RB form (supplemental Table S3).

##### Modulation of Trp Content as Defense Mechanism

As only few differences were observed between the protein profiles of the RB and ARB forms, we speculated if other factors could explain differences between the two phenotypes of *C. trachomatis*. It has been proposed that the level of host Trp available directly affects the bacterial protein profile (45). In general, proteins have a low content of Trp compared with other amino acids, but because of the physical and structural properties of Trp, the amino acid is important for protein folding and is often abundant in membrane proteins (46).

**Fig. 6.**
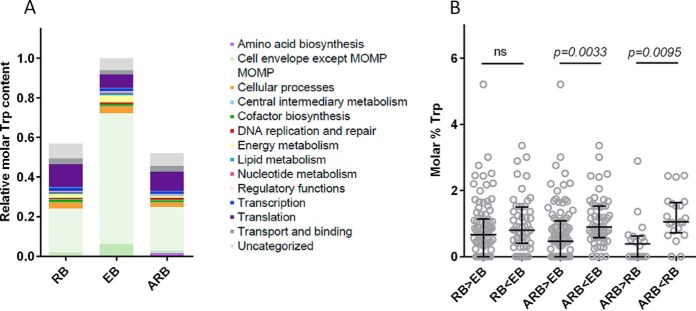
**The Trp content in the RB, EB and ARB growth forms.** *A*, The distribution of Trp among proteins grouped into different functional categories for each growth form; the total amount of tryptophan in the EB form was set to 1. The molar equivalents of the mean Trp content was calculated based on the iBAQ values for each of the biological replicates. MOMP belongs to the cell envelope but is here shown separately. *B*, Trp content in proteins which display changed abundance, the median values are shown. The comparison was performed by the Kruskal-Wallis test with Dunn's post test, and the comparisons were either nonsignificant (*ns*), or significant with the *p* values indicated.

At the genome level, the mean Trp content of *C. trachomatis* serovar D is 0.95% reflecting the relatively low abundance of this amino acid. The calculated mean Trp content of the identified proteins in RB, EB, and ARB samples was almost identical, 0.91, 0.91, and 0.89%, respectively, again reflecting that the majority of proteins are shared between the three samples. When calculating the total Trp content using the iBAQ values for each identified protein (supplemental Fig. S8), the content was found to be significantly higher in the EB form as compared with the RB and ARB form (1.76- and 1.92-fold, respectively), reflecting that the progression to EB depends on the availability of Trp and the EB form represents a “high-Trp state.” The distribution of the determined Trp content according to functional category was compared for all three growth forms, and as MOMP is a highly abundant protein, the Trp content of this specific protein is shown separately (Fig. 6*A*). Despite the fact that less Trp is allocated to proteins involved in translation in the EBs, the Trp needed for synthesis of cell envelope proteins, and in particular MOMP, was the single most important explanation for the increased demand of Trp in the EB form (almost 3-fold increase for this category alone). Each MOMP molecule contains 7 Trp residues (or 1.78% of the amino acid content) and with the strikingly high amount of MOMP in the EB form, the demand of Trp for MOMP (66% of the total Trp content) could explain the halt in expression of MOMP and thereby the halt in transition to EBs as previously hypothesized (47).

To further look for potential bias in the protein expression because of Trp availability, we compared the Trp content in the groups of proteins that display differential abundance (Fig. 6*B*). Comparison of the Trp content of proteins increased and decreased in RB compared with EB, showed no significant change. In contrast, we observed significant difference when we compared the Trp content in proteins increased in ARBs *versus* the proteins decreased in ARBs relative to both the EBs (*p* = 0.0033) and the RBs (*p* = 0.0095). This suggests, that *C. trachomatis* changes its protein profile by reduced expression of high-Trp proteins (here defined as above 1.5 molar % Trp) and thus adapts to the limited Trp availability. TrpA and TrpB are both low-Trp proteins (0% and 0.5% Trp, respectively), which allows the strongly increased expression of these proteins despite the Trp limitation. The TyrP_1 (Ct817) Trp transporter involved in Trp import from the host was also more abundant in the ARB form. Compared with other organisms, the *C. trachomatis* TyrP_1 transporter has a higher Trp content (2.8% Trp) (45), and its expression may be self-limiting as it will pose a Trp burden and therefore TyrP_1 may serve as a checkpoint for Trp import.

Our results experimentally confirm the hypothesis that *C. trachomatis* modulates the protein expression according to the Trp content (45, 48, 49). Under Trp limitation the protein repertoire is skewed toward expression of selected low-Trp proteins at the expense of the high-Trp proteins required to complete the normal developmental cycle. Thereby, the amount of Trp available for the bacterium and the Trp content of the individual proteins are important factors determining the proteome of the IFN-γ–induced persistence phenotype.

##### Conclusions and Perspectives

In the present study, we completed a quantitative proteome analysis of all *C. trachomatis* growth forms (EB, RB, and the IFN-γ induced ARB) for the *C. trachomatis* genital serovar D/UW-3/CX. We established an experimental approach that allowed comparison of this mixed-proteome in different growth phases. By the sensitive LC-MS/MS technique we identified 73% of the predicted proteins, of which 91 were not reported in previous proteome studies. The majority of the proteins (89%) were shared between the RB, EB, and ARB forms. The functional distribution of the proteins identified in each growth form was almost identical, emphasizing that the main differences between these phenotypes were because of differences in protein levels.

Quantitative analysis identified MOMP, Tuf, GroEL, and GroES as the most abundant proteins in all growth forms with MOMP constituting 13–39% of the total protein content dependent of the phenotype. A striking feature of the ARB phenotype, was the strongly increased abundance of the tryptophan synthase subunits TrpA and TrpB. These proteins appear to be markers of the persistence condition as confirmed by confocal microscopy and immunoblot analysis. Proteins increased in the ARBs, compared with the other growth forms, had a significantly lower Trp content *versus* proteins decreased in the ARBs, demonstrating that the Trp availability has a direct impact on the ARB protein repertoire.

Persistence represents a defense strategy for *C. trachomatis* to sense and escape immune control by the human host while maintaining viability and thus facilitating chronic infections. It is intriguing to speculate how this persistent *in vitro* phenotype is linked to the *in vivo* situation. Although a persistence-like phenotype has been described from a female patient (8), the precise role of persistent infections in Chlamydia genital tract disease has not yet been elucidated. Recently, reports of the role of IDO and Trp catabolism in persistent infections have accumulated for diverse pathogens of viral, bacterial, parasitic and fungal origin as reviewed (50). The *C. trachomatis in vitro* studies strongly suggest that this mechanism is also involved in chronic Chlamydia manifestations and demonstrate that TrpA and TrpB would be relevant to include as biomarkers for the persistence phenotype in further clinical studies.

Overall, our results provide insights in the function of the RB, EB and the IFN-γ induced ARB phenotypes and ultimately to the further development of diagnostics and interventions for Chlamydia infections.

## Supplementary Material

Supplemental Data

## References

[1] (2012) Global incidence and prevalence of selected curable sexually transmitted infections - 2008. pp. 1–28, WHO, Geneva, Switzerland

[2] Wyrick P. B. (2010) Chlamydia trachomatis Persistence In Vitro: An Overview. J. Infect. Dis. 201, S88–S95 20470046 10.1086/652394 PMC2878585

[3] Gérard H. C., Whittum-Hudson J. A., Schumacher H. R., and Hudson A. P. (2006) Synovial Chlamydia trachomatis up regulates expression of a panel of genes similar to that transcribed by Mycobacterium tuberculosis during persistent infection. Ann. Rheumatic Dis. 65, 321–327 10.1136/ard.2005.042226 PMC1798071 16192289

[4] Stephens R. S., Kalman S., Lammel C., Fan J., Marathe R., Aravind L., Mitchell W., Olinger L., Tatusov R. L., Zhao Q., Koonin E. V., and Davis R. W. (1998) Genome sequence of an obligate intracellular pathogen of humans: Chlamydia trachomatis. Science 282, 754–759 9784136 10.1126/science.282.5389.754

[5] Caldwell H. D., Wood H., Crane D., Bailey R., Jones R. B., Mabey D., Maclean I., Mohammed Z., Peeling R., Roshick C., Schachter J., Solomon A. W., Stamm W. E., Suchland R. J., Taylor L., West S. K., Quinn T. C., Belland R. J., and McClarty G. (2003) Polymorphisms in Chlamydia trachomatis tryptophan synthase genes differentiate between genital and ocular isolates. J. Clin. Invest. 111, 1757–1769 12782678 10.1172/JCI17993 PMC156111

[6] Beatty W. L., Morrison R. P., and Byrne G. I. (1995) Reactivation of persistent Chlamydia trachomatis infection in cell culture. Infect. Immun. 63, 199–205 7806358 10.1128/iai.63.1.199-205.1995 PMC172978

[7] Belland R. J., Nelson D. E., Virok D., Crane D. D., Hogan D., Sturdevant D., Beatty W. L., and Caldwell H. D. (2003) Transcriptome analysis of chlamydial growth during IFN-gamma-mediated persistence and reactivation. Proc. Natl. Acad. Sci. U.S.A. 100, 15971–15976 14673075 10.1073/pnas.2535394100 PMC307677

[8] Lewis M. E., Belland R. J., Abdelrahman Y. M., Beatty W., Aiyar A. A., Zea A. H., Greene S. J., Marrero L., Buckner L. R., Tate D. J., McGowin C. L., Kozlowski P. A., O'Brien M., Lillis R. A., Martin D. H., and Quayle A. J. (2014) Morphologic and molecular evaluation of Chlamydia trachomatis growth in human endocervix reveals distinct growth patterns. Front. Cell Infect. Microbiol. 4, 71 24959423 10.3389/fcimb.2014.00071 PMC4050528

[9] Hogan R. J., Mathews S. A., Mukhopadhyay S., Summersgill J. T., and Timms P. (2004) Chlamydial persistence: beyond the biphasic paradigm. Infect Immun 72, 1843–1855 15039303 10.1128/IAI.72.4.1843-1855.2004 PMC375192

[10] Vandahl B. B., Birkelund S., Demol H., Hoorelbeke B., Christiansen G., Vandekerckhove J., and Gevaert K. (2001) Proteome analysis of the Chlamydia pneumoniae elementary body. Electrophoresis 22, 1204–1223 11358148 10.1002/1522-2683()22:6<1204::AID-ELPS1204>3.0.CO;2-M

[11] Wehrl W., Meyer T. F., Jungblut P. R., Muller E. C., and Szczepek A. J. (2004) Action and reaction: Chlamydophila pneumoniae proteome alteration in a persistent infection induced by iron deficiency. Proteomics 4, 2969–2981 15378754 10.1002/pmic.200400917

[12] Skipp P., Robinson J., O'Connor C. D., and Clarke I. N. (2005) Shotgun proteomic analysis of Chlamydia trachomatis. Proteomics 5, 1558–1573 15838905 10.1002/pmic.200401044

[13] Saka H. A., Thompson J. W., Chen Y. S., Kumar Y., Dubois L. G., Moseley M. A., and Valdivia R. H. (2011) Quantitative proteomics reveals metabolic and pathogenic properties of Chlamydia trachomatis developmental forms. Mol. Microbiol. 82, 1185–1203 22014092 10.1111/j.1365-2958.2011.07877.x PMC3225693

[14] Skipp P. J., Hughes C., McKenna T., Edwards R., Langridge J., Thomson N. R., and Clarke I. N. (2016) Quantitative Proteomics of the Infectious and Replicative Forms of Chlamydia trachomatis. PLoS ONE 11, e0149011 26871455 10.1371/journal.pone.0149011 PMC4752267

[15] Olsen A. W., Follmann F., Erneholm K., Rosenkrands I., and Andersen P. (2015) Protection Against Chlamydia trachomatis Infection and Upper Genital Tract Pathological Changes by Vaccine-Promoted Neutralizing Antibodies Directed to the VD4 of the Major Outer Membrane Protein. J. Infect. Dis. 212, 978–989 25748320 10.1093/infdis/jiv137

[16] Scidmore M. A. (2005) Cultivation and Laboratory Maintenance of Chlamydia trachomatis. Curr. Protoc. Microbiol. **Chapter** 11, Unit 11A 11 10.1002/9780471729259.mc11a01s00 18770550

[17] Albrethsen J., Agner J., Piersma S. R., Hojrup P., Pham T. V., Weldingh K., Jimenez C. R., Andersen P., and Rosenkrands I. (2013) Proteomic profiling of Mycobacterium tuberculosis identifies nutrient-starvation-responsive toxin-antitoxin systems. Mol. Cell. Proteomics 12, 1180–1191 23345537 10.1074/mcp.M112.018846 PMC3650330

[18] Shevchenko A., Tomas H., Havlis J., Olsen J. V., and Mann M. (2006) In-gel digestion for mass spectrometric characterization of proteins and proteomes. Nat. Protoc. 1, 2856–2860 17406544 10.1038/nprot.2006.468

[19] Rappsilber J., Ishihama Y., and Mann M. (2003) Stop and go extraction tips for matrix-assisted laser desorption/ionization, nanoelectrospray, and LC/MS sample pretreatment in proteomics. Anal. Chem. 75, 663–670 12585499 10.1021/ac026117i

[20] Cox J., and Mann M. (2008) MaxQuant enables high peptide identification rates, individualized p.p.b.-range mass accuracies and proteome-wide protein quantification. Nat. Biotechnol. 26, 1367–1372 19029910 10.1038/nbt.1511

[21] Cox J., Neuhauser N., Michalski A., Scheltema R. A., Olsen J. V., and Mann M. (2011) Andromeda: a peptide search engine integrated into the MaxQuant environment. J. Proteome Res. 10, 1794–1805 21254760 10.1021/pr101065j

[22] Schwanhausser B., Busse D., Li N., Dittmar G., Schuchhardt J., Wolf J., Chen W., and Selbach M. (2011) Global quantification of mammalian gene expression control. Nature 473, 337–342 21593866 10.1038/nature10098

[23] Elias J. E., and Gygi S. P. (2007) Target-decoy search strategy for increased confidence in large-scale protein identifications by mass spectrometry. Nat. Methods 4, 207–214 17327847 10.1038/nmeth1019

[24] Li Z., Chen C., Chen D., Wu Y., Zhong Y., and Zhong G. (2008) Characterization of fifty putative inclusion membrane proteins encoded in the Chlamydia trachomatis genome. Infect. Immun. 76, 2746–2757 18391011 10.1128/IAI.00010-08 PMC2423075

[25] Lutter E. I., Martens C., and Hackstadt T. (2012) Evolution and conservation of predicted inclusion membrane proteins in chlamydiae. Comp. Funct. Genomics 2012, 362104 22454599 10.1155/2012/362104 PMC3290821

[26] Peters J., Wilson D. P., Myers G., Timms P., and Bavoil P. M. (2007) Type III secretion a la Chlamydia. Trends Microbiol. 15, 241–251 17482820 10.1016/j.tim.2007.04.005

[27] Spaeth K. E., Chen Y. S., and Valdivia R. H. (2009) The Chlamydia type III secretion system C-ring engages a chaperone-effector protein complex. PLoS Pathog. 5, e1000579 19750218 10.1371/journal.ppat.1000579 PMC2734247

[28] Weber M. M., Bauler L. D., Lam J., and Hackstadt T. (2015) Expression and Localization of Predicted Inclusion Membrane Proteins in Chlamydia trachomatis. Infect. Immun. 83, 4710–4718 26416906 10.1128/IAI.01075-15 PMC4645406

[29] Miyairi I., Mahdi O. S., Ouellette S. P., Belland R. J., and Byrne G. I. (2006) Different growth rates of Chlamydia trachomatis biovars reflect pathotype. J. Infect. Dis. 194, 350–357 16826483 10.1086/505432

[30] Shaw A. C., Christiansen G., Roepstorff P., and Birkelund S. (2000) Genetic differences in the Chlamydia trachomatis tryptophan synthase alpha-subunit can explain variations in serovar pathogenesis. Microbes Infect. 2, 581–592 10884608 10.1016/s1286-4579(00)00368-3

[31] Washburn M. P., Wolters D., and Yates J. R. 3rd. (2001) Large-scale analysis of the yeast proteome by multidimensional protein identification technology. Nat. Biotechnol. 19, 242–247 11231557 10.1038/85686

[32] Issaq H., and Veenstra T. (2008) Two-dimensional polyacrylamide gel electrophoresis (2D-PAGE): advances and perspectives. BioTechniques 44, 697–698, 700 18474047 10.2144/000112823

[33] Hackstadt T., and Caldwell H. D. (1985) Effect of proteolytic cleavage of surface-exposed proteins on infectivity of Chlamydia trachomatis. Infect. Immun. 48, 546–551 2580794 10.1128/iai.48.2.546-551.1985 PMC261371

[34] Adams N. E., Thiaville J. J., Proestos J., Juarez-Vazquez A. L., McCoy A. J., Barona-Gomez F., Iwata-Reuyl D., de Crecy-Lagard V., and Maurelli A. T. (2014) Promiscuous and adaptable enzymes fill “holes” in the tetrahydrofolate pathway in Chlamydia species. MBio 5, e01378–e01314 25006229 10.1128/mBio.01378-14 PMC4161248

[35] Belland R. J., Zhong G., Crane D. D., Hogan D., Sturdevant D., Sharma J., Beatty W. L., and Caldwell H. D. (2003) Genomic transcriptional profiling of the developmental cycle of Chlamydia trachomatis. Proc. Natl. Acad. Sci. U.S.A. 100, 8478–8483 12815105 10.1073/pnas.1331135100 PMC166254

[36] Rosario C. J., and Tan M. (2012) The early gene product EUO is a transcriptional repressor that selectively regulates promoters of Chlamydia late genes. Mol. Microbiol. 84, 1097–1107 22624851 10.1111/j.1365-2958.2012.08077.x PMC3544401

[37] Kari L., Southern T. R., Downey C. J., Watkins H. S., Randall L. B., Taylor L. D., Sturdevant G. L., Whitmire W. M., and Caldwell H. D. (2014) Chlamydia trachomatis polymorphic membrane protein D is a virulence factor involved in early host-cell interactions. Infect. Immun. 82, 2756–2762 24733093 10.1128/IAI.01686-14 PMC4097629

[38] Omsland A., Sager J., Nair V., Sturdevant D. E., and Hackstadt T. (2012) Developmental stage-specific metabolic and transcriptional activity of Chlamydia trachomatis in an axenic medium. Proc. Natl. Acad. Sci. U.S.A. 109, 19781–19785 23129646 10.1073/pnas.1212831109 PMC3511728

[39] Fields K. A., Mead D. J., Dooley C. A., and Hackstadt T. (2003) Chlamydia trachomatis type III secretion: evidence for a functional apparatus during early-cycle development. Mol. Microbiol. 48, 671–683 12694613 10.1046/j.1365-2958.2003.03462.x

[40] Budrys N. M., Gong S., Rodgers A. K., Wang J., Louden C., Shain R., Schenken R. S., and Zhong G. (2012) Chlamydia trachomatis antigens recognized in women with tubal factor infertility, normal fertility, and acute infection. Obstet. Gynecol. 119, 1009–1016 22525912 10.1097/AOG.0b013e3182519326 PMC4608258

[41] Toye B., Laferriere C., Claman P., Jessamine P., and Peeling R. (1993) Association between antibody to the chlamydial heat-shock protein and tubal infertility. J. Infect. Dis. 168, 1236–1240 7901289 10.1093/infdis/168.5.1236

[42] Beatty W. L., Byrne G. I., and Morrison R. P. (1993) Morphologic and antigenic characterization of interferon gamma-mediated persistent Chlamydia trachomatis infection in vitro. Proc. Natl. Acad. Sci. U.S.A. 90, 3998–4002 8387206 10.1073/pnas.90.9.3998 PMC46433

[43] Gerard H. C., Freise J., Wang Z., Roberts G., Rudy D., Krauss-Opatz B., Kohler L., Zeidler H., Schumacher H. R., Whittum-Hudson J. A., and Hudson A. P. (2002) Chlamydia trachomatis genes whose products are related to energy metabolism are expressed differentially in active vs. persistent infection. Microbes Infect. 4, 13–22 11825770 10.1016/s1286-4579(01)01504-0

[44] Ouellette S. P., Hatch T. P., AbdelRahman Y. M., Rose L. A., Belland R. J., and Byrne G. I. (2006) Global transcriptional upregulation in the absence of increased translation in Chlamydia during IFNgamma-mediated host cell tryptophan starvation. Mol. Microbiol. 62, 1387–1401 17059564 10.1111/j.1365-2958.2006.05465.x

[45] Lo C. C., Xie G., Bonner C. A., and Jensen R. A. (2012) The alternative translational profile that underlies the immune-evasive state of persistence in Chlamydiaceae exploits differential tryptophan contents of the protein repertoire. Microbiol. Mol. Biol. Rev. 76, 405–443 22688818 10.1128/MMBR.05013-11 PMC3372251

[46] Schiffer M., Chang C. H., and Stevens F. J. (1992) The functions of tryptophan residues in membrane proteins. Protein Eng. 5, 213–214 1409540 10.1093/protein/5.3.213

[47] Beatty W. L., Belanger T. A., Desai A. A., Morrison R. P., and Byrne G. I. (1994) Tryptophan depletion as a mechanism of gamma interferon-mediated chlamydial persistence. Infect. Immun. 62, 3705–3711 8063385 10.1128/iai.62.9.3705-3711.1994 PMC303021

[48] Bonner C. A., Byrne G. I., and Jensen R. A. (2014) Chlamydia exploit the mammalian tryptophan-depletion defense strategy as a counter-defensive cue to trigger a survival state of persistence. Front. Cell Infect. Microbiol. 4, 17 24616884 10.3389/fcimb.2014.00017 PMC3937554

[49] Huston W. M., Barker C. J., Chacko A., and Timms P. (2014) Evolution to a chronic disease niche correlates with increased sensitivity to tryptophan availability for the obligate intracellular bacterium Chlamydia pneumoniae. J. Bacteriol. 196, 1915–1924 24682324 10.1128/JB.01476-14 PMC4010988

[50] Barth H., and Raghuraman S. (2014) Persistent infectious diseases say - IDO. Role of indoleamine-2,3-dioxygenase in disease pathogenesis and implications for therapy. Crit. Rev. Microbiol. 40, 360–368 23174025 10.3109/1040841X.2012.742037

