# Supplementary material for: Quantitative Protein Profiling of Chlamydia trachomatis Growth Forms Reveals Defense Strategies Against Tryptophan Starvation
Source: Mol Cell Proteomics. 2016 Oct 26;15(12):3540–50. doi: 10.1074/mcp.M116.061986 (PMC5141270; doi:10.1074/mcp.M116.061986)
Supplement: Supplemental Data [file supp_15_12_3540__index.html]

Quantitative protein profiling of Chlamydia trachomatis growth forms reveals defense strategies against tryptophan starvation — Quantitative Protein Profiling of Chlamydia trachomatis Growth Forms Reveals Defense Strategies Against Tryptophan Starvation — The Chlamydia Proteome Under Tryptophan Starvation — Supplemental Data 

# Quantitative Protein Profiling of *Chlamydia trachomatis* Growth Forms Reveals Defense Strategies Against Tryptophan Starvation

## Supplemental Data

- Supplemental Data 1 (.xlsx, 21.4 MB) - Supplemental Data 1
- Supplemental Table 1 (.xlsx, 509 KB) - Supplemental Table 1
- Supplemental Table 2 (.xlsx, 599 KB) - Supplemental Table 2
- Supplemental Table 3 (.pdf, 83 KB) - Supplemental Table 3
- Supplemental Figures 1-8 (.pdf, 938 KB) - Supplemental Figures 1-8
