# Supplementary material for: Quantitative Protein Profiling of Chlamydia trachomatis Growth Forms Reveals Defense Strategies Against Tryptophan Starvation
Source: Mol Cell Proteomics. 2016 Oct 26;15(12):3540–50. doi: 10.1074/mcp.M116.061986 (PMC5141270; doi:10.1074/mcp.M116.061986)
Supplement: Supplemental Data [file 10.1074_M116.061986_mcp.M116.061986-4.pdf]

Supplemental Table 3. Functional enrichment of differentially abundant proteins. Significantly enriched (*Fisher's* exact p-value <0.05) functional categories are indicated.

Functional category	Proteins	Changed in category	Total in category	Fold enrichment	Fisher's exact *p*-value
**RB>EB**					
Translation[a]	RplA, RplB, RplD, RplE, RplF, RplI, RplJ, RplK, RplL, RplM, RplN, RplO, RplP, RplQ, RplR, RplT, RplW, RplY, RpmE2, RpsD, RpsE, RpsG, RpsI, RpsM, RpsP, Efp2, InfC, LeuS, Rrf, Tsf	30	108	2.0	$<10^{-4}$
Inclusion membrane proteins[b]	IncA, IncG, Ct147, Ct223, Ct228, Ct229, Ct249, Ct618	8	21	2.7	0.0051
**RB<EB**					
Cell envelope. Membranes, lipoproteins and porins[a]	MOMP, OmcB, OmpB, PmpB, PmpE, PmpF, PmpG, PmpH	8	18	5.5	$<10^{-4}$
Carbohydrate metabolism[c]	FbaB, GlgP, PckG, Pgi, Pgk, Pyk, SucA, Tal	8	45	2.2	0.0209
Structural components of T3SS[d]	CdsC, CdsD, CdsF, CdsJ, CdsQ, CdsV, CopB	7	13	6.7	$<10^{-4}$
**ARB>EB**					
Translation[a]	RplA, RplC, RplD, RplE, RplF, RplI, RplJ, RplK, RplL, RplM, RplN, RplO, RplP, RplQ, RplR, RplT, RplW, RplY, RpmE2, RpsD, RpsE, RpsG, RpsI, RpsM, RpsP, HisS, InfC, Mip	28	108	1.7	0.0011
Inclusion membrane proteins[b]	IncA, IncG, Ct147, Ct223, Ct228, Ct229, Ct249, Ct529, Ct618	9	21	2.8	0.0019
**ARB<EB**					
Cell envelope. Membranes, lipoproteins and porins[a]	MOMP, OmcB, OmpB, PmpB, PmpE, PmpF, PmpG, PmpH	8	18	5.0	$<10^{-4}$
Carbohydrate metabolism[c]	FbaB, Gap, GlgP, Gnd, Mdh, PckG, PdhC, Pgi, Pyk, SucA, Tal	11	45	2.8	0.0009
Structural components of T3SS[d]	CdsC, CdsD, CdsF, CdsJ, CdsQ, CdsV, CopB	7	13	6.1	$<10^{-4}$
**ARB>RB**					
No enriched categories identified					
**ARB<RB**					
No enriched categories identified					

[a]Functional category according to http://stdgen.northwestern.edu/

[b]Functional annotation based on literature (24, 25, 28)

[c]Functional category according to KEGG http://www.genome.jp/kegg/

[d]Functional annotation based on literature (26, 27)
