# Supplementary material for: Quantitative Protein Profiling of Chlamydia trachomatis Growth Forms Reveals Defense Strategies Against Tryptophan Starvation
Source: Mol Cell Proteomics. 2016 Oct 26;15(12):3540–50. doi: 10.1074/mcp.M116.061986 (PMC5141270; doi:10.1074/mcp.M116.061986)
Supplement: Supplemental Data [file 10.1074_M116.061986_mcp.M116.061986-5.pdf]

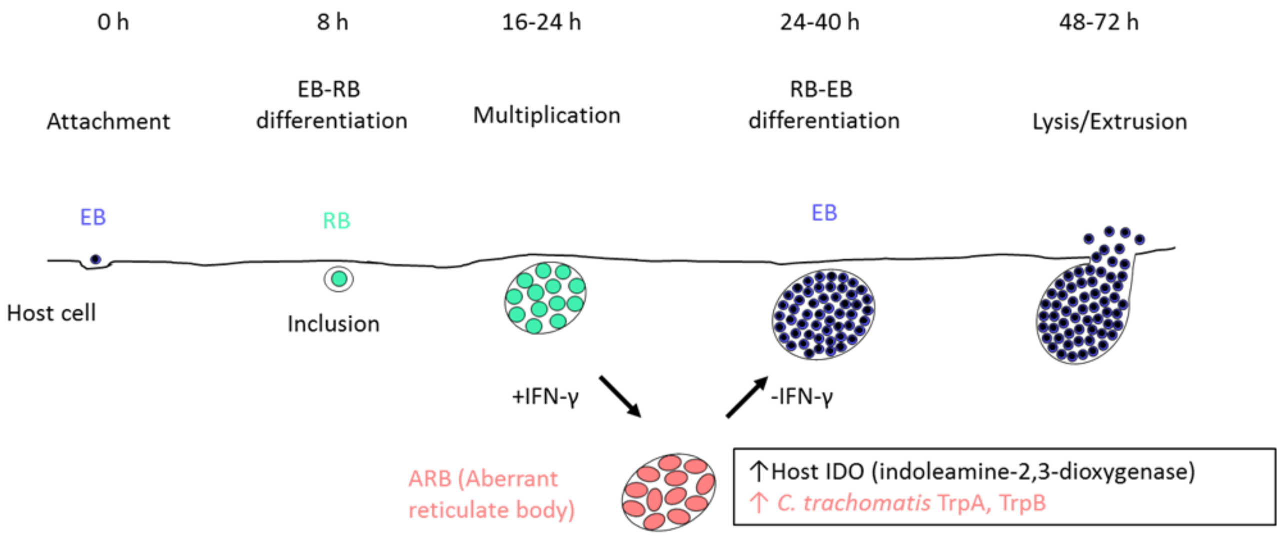

Supplemental Figure 1. Depiction of the chlamydial growth forms with approximate time points for the genital strains. The infection is initiated with attachment of an elementary body (EB), that is internalized. The EB ends up in the inclusion which is a membrane bound vacuole. Subsequently, the EB differentiates to the RB (reticulate body) form, and RB multiplication by binary fission takes place. RBs then differentiate back to the EB form which is released from the host cell by lysis or extrusion.

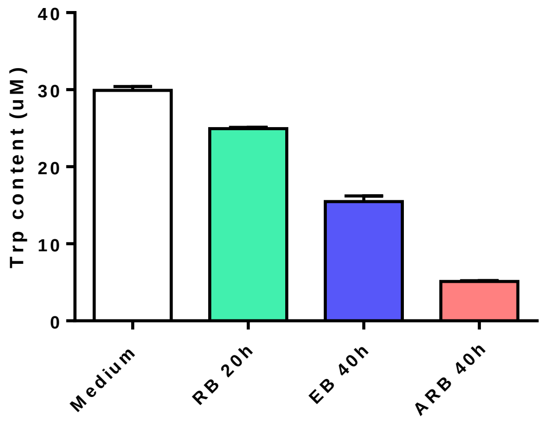

Supplemental Figure 2. The tryptophan (Trp) content was determined in the spent cell culture medium harvested from the RBs, EBs and ARBs at the selected time points. Mean Trp values ± SD are shown from triplicate cultures. The Trp content in the medium before culturing is shown for comparison.

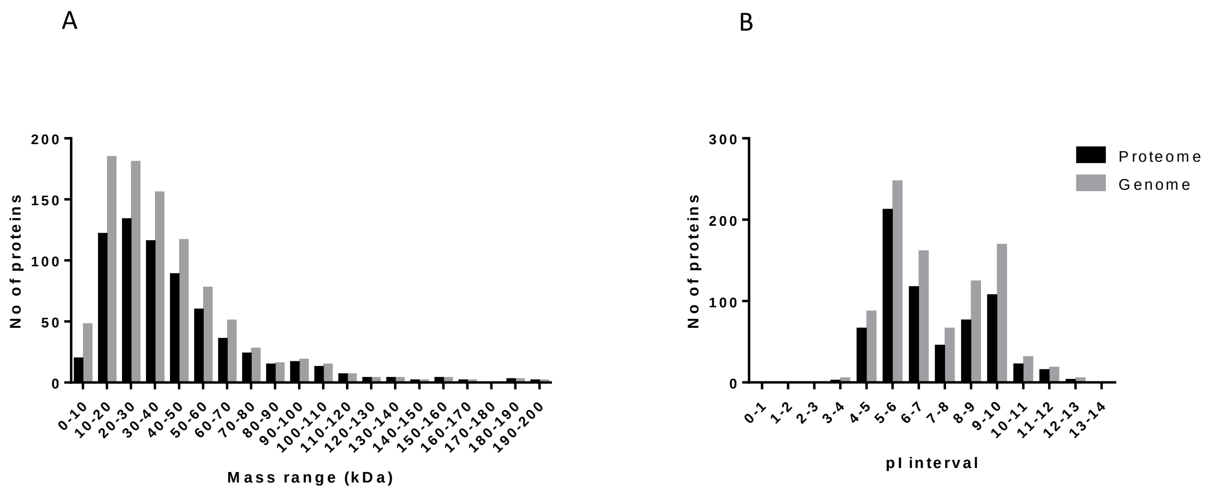

Supplemental Figure 3. Distribution of the identified proteins according to molecular mass and pI. Mass (A) and pI (B) values of all identified proteins (black bars) are shown, and values of all predicted proteins from the genome sequence are shown for comparison (grey bars). Mass and pI values were calculated by the algorithm available at http://web.expasy.org/compute_pi/.

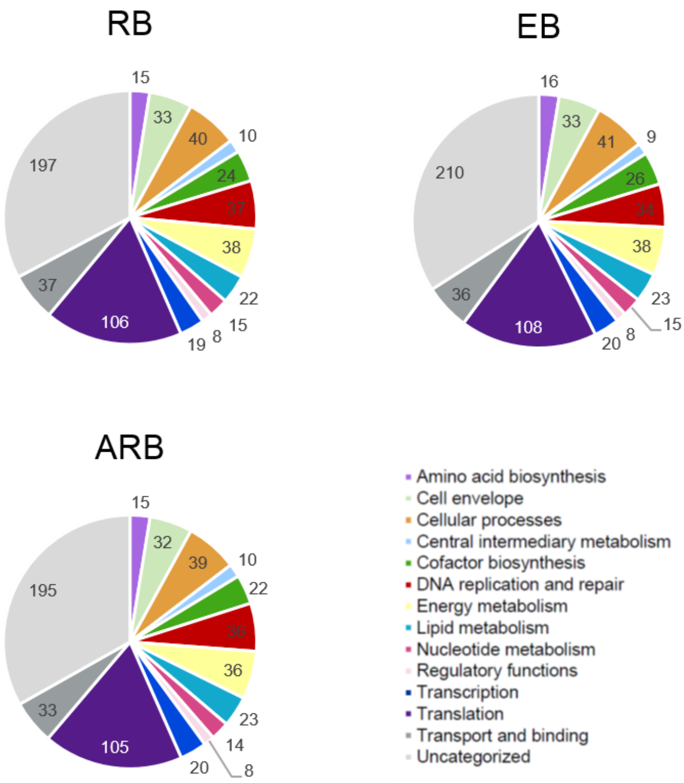

Supplemental Figure 4. Distribution of identified proteins in functional groups in the RB, EB and ARB forms. The number of proteins belonging to each category are shown.

A

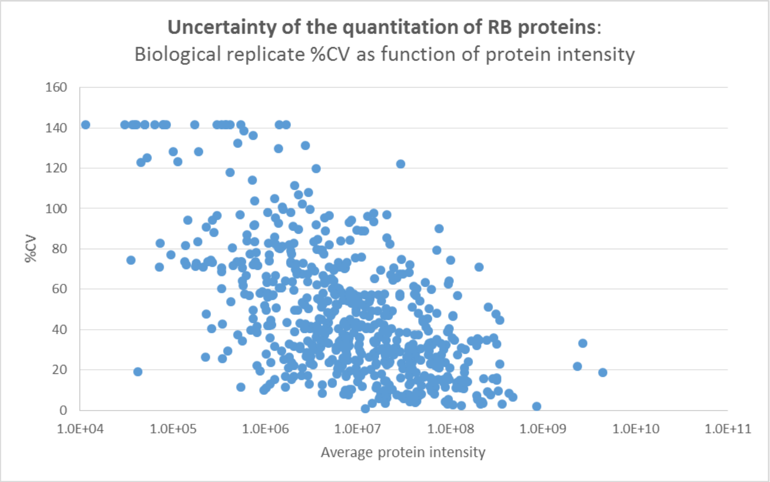

B

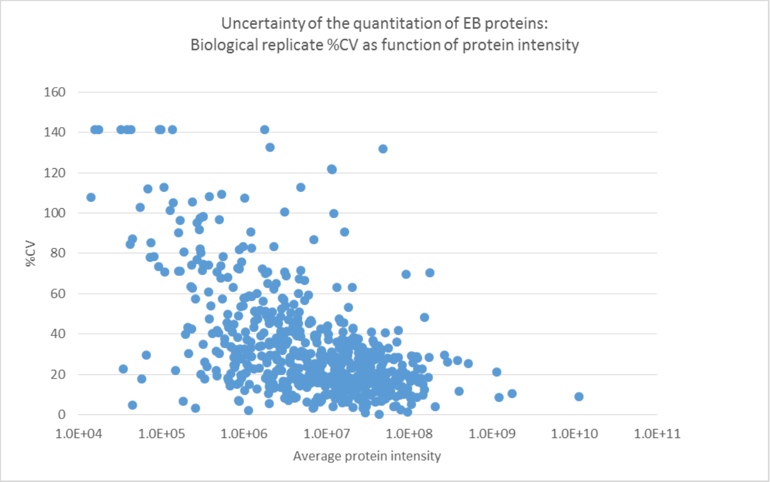

C

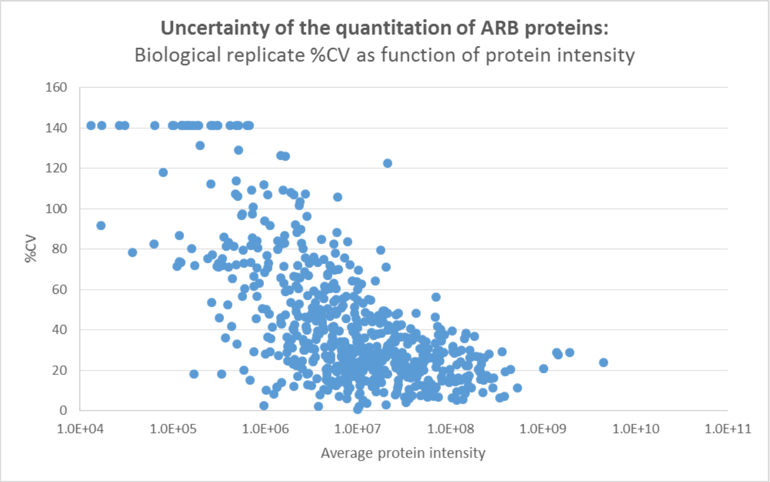

Supplemental Figure 5. Distribution of protein-level CV's of normalized MS1 intensities for the biological replicates of each growth form RB (A), EB (B) and ARB (C).

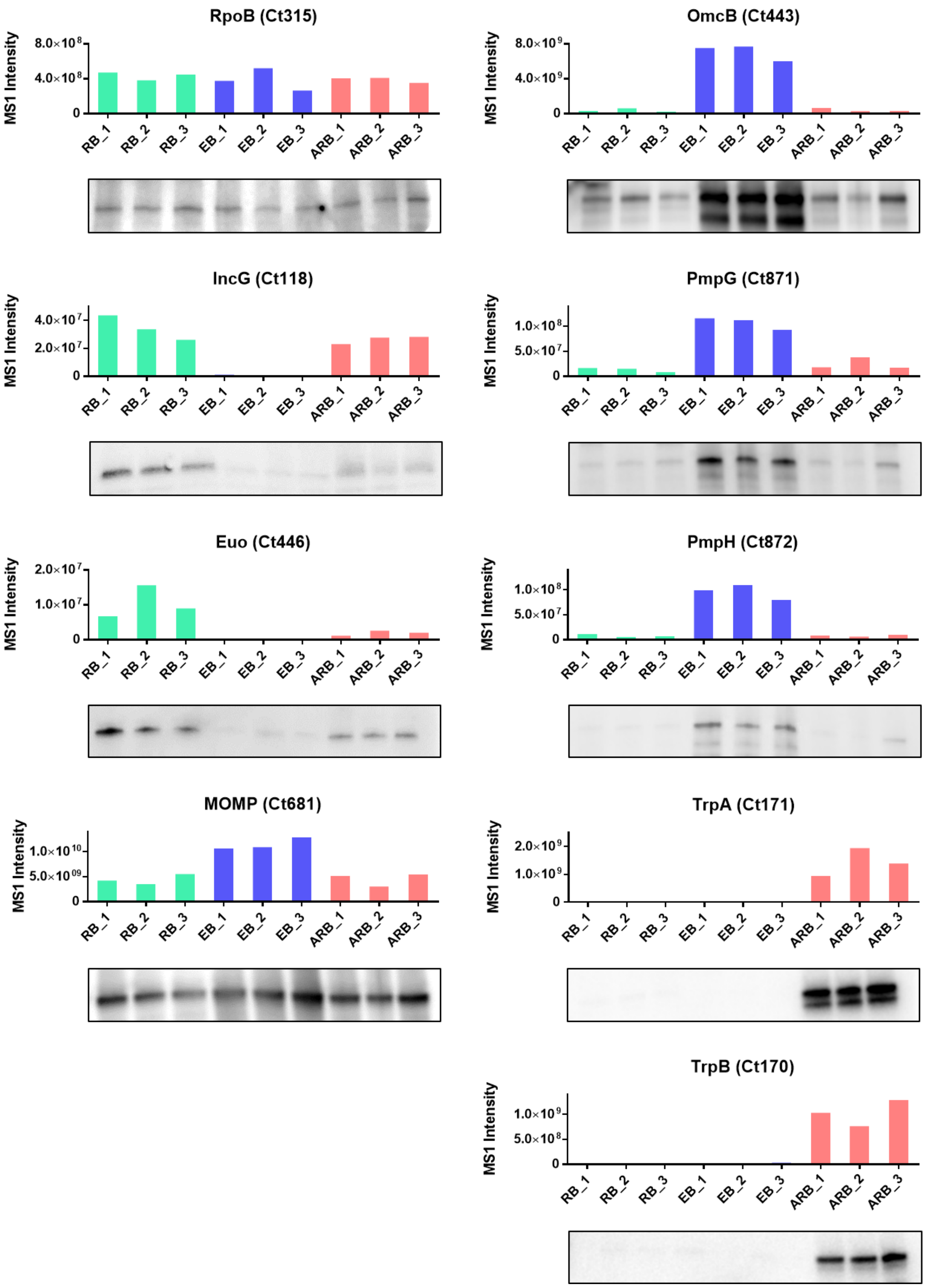

Supplemental Figure 6. Validation of proteomics MS analysis by immunoblot analysis. For each protein the upper section shows the normalized MS1 intensity, and the lower section shows the corresponding sample immunoblot.

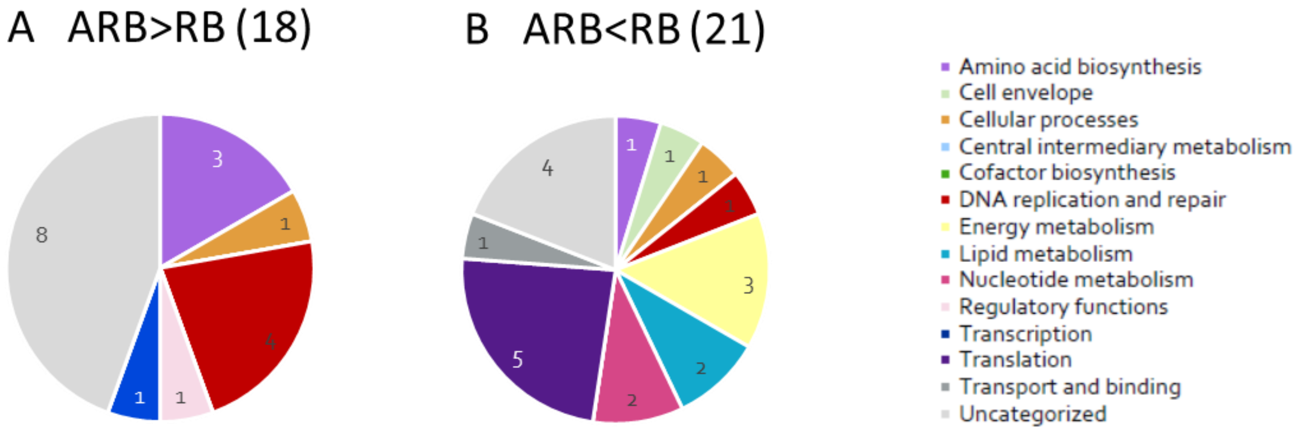

Supplemental Figure 7. Comparison of proteins with differential abundance in the RB and ARB forms. Pie chart representing functional categorization of (A) ARB increased proteins and (B) ARB decreased proteins compared to RB. The number of proteins belonging to each category are shown.

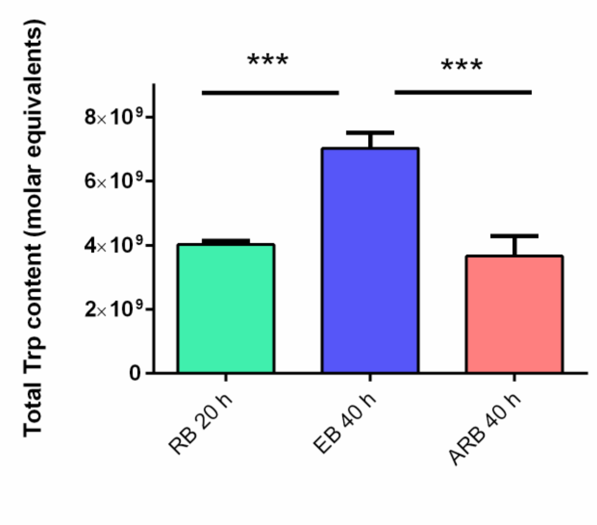

Supplemental Figure 8. Trp content of each growth form calculated based on the iBAQ values. Mean values ± SD are shown from triplicate samples. Comparisons were performed by ANOVA analysis with Tukey's post test, $p<0.001$ (***).
